# Miscellany

**Published:** 1894-02

**Authors:** 


### Miscellany.

An Army Medical Board will be in session at Washington. D. C., during April, 1894, for the examination of candidates for appointment to the medical corps of the United States Army, to fill existing vacancies.

Persons desiring to present themselves for examination by the Board will make application to the Secretary of War before March 15, 1894, for the necessary invitation, giving the date and place of birth, the place and State of permanent residence, the fact of American citizenship, the name of the medical college from which they were graduated, and a record of service in hospital, if any, from the authorities thereof. The application should be accompanied by certificates, based on personal acquaintance, from at least two reputable persons, as to his citizenship, character and habits. The candidate must be between 22 and 28 years of age, and a graduate from a regular medical college, as evidence of which, his diploma must be submitted to the Board. Successful candidates at the coming examination will be given a course of instruction at the next session of the Army Medical School, beginning in November, 1894. Further information regarding the examinations may be obtained by addressing the Surgeon-General, U. S. Army, Washington, D. C.

